# Neuronal epigenetics and the aging synapse

**DOI:** 10.3389/fncel.2015.00208

**Published:** 2015-05-27

**Authors:** Jorge Azpurua, Benjamin A. Eaton

**Affiliations:** Department of Physiology, University of Texas Health Science Center at San AntonioSan Antonio, TX, USA

**Keywords:** aging, epigenetics, synapses, acetylation, neurotransmission

## Abstract

Two of the most salient phenotypes of aging are cognitive decline and loss of motor function, both of which are controlled by the nervous system. Cognition and muscle contraction require that neuronal synapses develop and maintain proper structure and function. We review the literature on how normal physiological aging disrupts central and peripheral synapse function including the degradation of structure and/or control of neurotransmission. Here we also attempt to connect the work done on the epigenetics of aging to the growing literature of how epigenetic mechanisms control synapse structure and function. Lastly, we address possible roles of epigenetic mechanisms to explain why the basal rates of age-related dysfunction vary so widely across individuals.

## Introduction

Synapses are the functional unit that processes signals between neurons, and between an innervating motor neuron and its target muscle. Neurotransmitter release at the synapse is required for planning and executive function, memory formation and recall, motor behavior, hormone release, and virtually every organismal function that feeds back into the central nervous system. Modulation of synaptic strength is heavily implicated in the persistence of memory and general cognitive functions. The synapse is a particularly interesting target for the effects of aging on nervous system function. The structure of the synapse is complex and many synapses must be maintained throughout the life of the animal. Additionally, the composition of ion channels at both pre- and post synaptic terminals is highly heterogeneous, crucial for function, and may cause synapse malfunction if altered. Lastly, synaptic dysfunction could affect neuroendocrine signaling. Coordination of synaptic vesicle release requires the neuron to maintain proper synaptic structure, membrane excitability and neurotransmission, as well as integrate retrograde signals from the post-synaptic terminal. Defects in these complex processes have been implicated in many diseases including Alzheimer’s disease (AD), and Parkinson’s disease (PD), which both feature a strong aging-associated risk component (Hebert et al., 2001; Levy et al., 2002).

Here we review the growing literature on the contribution of one hallmark of cellular aging—epigenetic alterations- on neuronal aging, with a strict focus on synaptic structure and function during non-pathological aging. The effects of adult onset neurodegenerative disease on synapse function have been extensively reviewed elsewhere (Wishart et al., 2006; Gillingwater and Wishart, 2013). We use “epigenetics” broadly referring to stable and heritable changes in gene expression that can be transmitted across cell divisions without DNA mutations. Major mechanisms of epigenetic control include DNA CpG methylation, histone modifications (acetylation and methylation), deposition of alternative histones, and RNA silencing. Aging organisms undergo genome-wide DNA CpG demethylation across their tissues, leading to wide transcriptional induction. Histone methylation and acetylation have also been reported to change with age (Calvanese et al., 2009). These changes are associated with various disease states, such as a cancer (Esteller et al., 2001). Proper synaptic function requires tight co-regulation of many genes, making epigenetic dysfunction an attractive candidate to explain age-associated declines in the nervous system. The growing recognition of neuroepigenetics has been previously reviewed (Sweatt, 2013). Here we will attempt to link what is known about epigenetic regulation in the nucleus, to non-pathological aging-related dysfunction of the synaptic terminal.

## Synaptic Structure and Aging : Central and Sensory Synapses

### Central Synapse Structure is Altered by Aging

A substantial body of evidence supports the idea that aging disrupts synaptic connections in the central nervous system. Synapses may be completely lost in some contexts and the evidence strongly points at synaptic loss being a key feature of general brain aging as well as a hallmark of AD pathology (DeKosky and Scheff, 1990; Cabalka et al., 1992; Masliah et al., 1993; Scheff and Price, 2001; Coleman and Yao, 2003). In humans, there is support for an association between age related mild-cognitive impairment and loss of synapses and there is evidence in rodents as well (Coggan et al., 2004; Scheff et al., 2006; Canas et al., 2009; Richard et al., 2010). In some tissues, loss of postsynaptic dendritic spines has been implicated as the primary mechanism of synapse loss (Feldman and Dowd, 1975; Geinisman et al., 1992; de Brabander et al., 1998). Changes in synaptic structure and synapse loss with age by neuroanatomical region have been thoroughly reviewed (Petralia et al., 2014) and the evidence is robust. A recent study found that age-dependent declines in cognition and memory may best be explained as a decline in synaptic stability (Grillo et al., 2013). Grillo and colleagues used two-photon microscopy and semi-automated image processing to analyze synapse structure across age. They found that as age increases, there is a loss of “synaptic tenacity” driven by increases in the rate of bouton loss and remodeling.

Age-dependent changes to synapse structure have been studied in other model organisms as well. In *C. elegans*, animals show an age-dependent increase in a number of dendritic defects of sensory neurons, including ectopic branch points, which is predicted to alter synaptic connectivity (Toth et al., 2012). Intriguingly, in *Drosophila* sensory neurons the change is opposite of that seen in *C. elegans*, with fewer branch points as age increases (Corfas and Dudai, 1991). Thus the synapse may be an Achilles’ heel that is vulnerable to aging across all metazoan taxa.

### Histone Acetylation and Central Synapse Structure

New evidence suggests that epigenetic alterations during aging may be driving central synapse loss and alterations in structure. Modulating the expression level of histone deacetylase 2 (HDAC2) has been shown to cause changes in synaptic structure of the CA1 pyramidal neurons of the mouse hippocampus (Guan et al., 2009). Overexpression of HDAC2 led to a reduction in the number of spines as seen through Golgi staining and decreased synaptophysin staining, whereas HDAC2 knockout showed an increase in both. HDAC2 is a class 1 (NAD-independent) histone deacetylase, which generally acts as a transcriptional silencer in concert with YY1 (Yang et al., 1996) and DNA methyl transferase 1 (DMNT1; Rountree et al., 2000). Chromatin immunoprecipitation from whole mouse brains of HDAC2 and HDAC1 both showed enrichment for cell cycle genes, but HDAC2 also showed higher enrichment of genes involved in synaptic formation and plasticity (e.g., *Nrxn3* and *Synapsin2*). This report strongly supports the involvement of HDAC2 in structural synaptic maintenance.

## Synaptic Structure and Aging: Neuromuscular Junction

### Neuromuscular Junction Synapse Structure is Altered by Aging

Motor decline is one the most predictive biomarkers of mortality risk in humans (Buchman et al., 2007). Age-related pathologies in the neuromuscular junction (NMJ) have been studied in rodents since the early 1970’s (Gutmann et al., 1971; Banker et al., 1983; Cardasis and LaFontaine, 1987). More recently, live imaging of SOD1 knock-out mice sternomastoid muscles revealed a pronounced retraction of the NMJ with over two-thirds of NMJs denervated, recapitulating the aging phenotype in an accelerated fashion (Jang et al., 2010). Conversely, caloric restriction (a conserved pathway known to slow aging rates) was found to also abrogate the denervation of mouse NMJs (Valdez et al., 2010). Nonetheless, aging does not seem to affect all motor neurons equally, as brainstem motor neurons are at least partially resistant to age-dependent denervation (Valdez et al., 2012). Synapse morphology may also be altered in response to muscle fiber loss and regeneration (Li et al., 2011). It is possible that fiber loss is followed by improper synapse innervations due to compromised synaptogenesis in aged neurons.

The NMJ synapses in both larval and adult *Drosophila* have been characterized with development and aging as well. Initial imaging studies in the longitudinal abdominal muscle of adult flies showed several age-dependent structural changes, including enlarged bouton area, decreased branch length and “naked boutons” directly facing the extracellular basal lamina (Beramendi et al., 2007). In the same study, the authors also showed a significant increase in synaptic vesicle size, as well as changes in mitochondrial structure and area. Together with mammalian studies, this supports that alterations to the NMJ structure are a conserved aging phenotype.

As with central synapses, there is evidence to support the involvement of epigenetic factors in age-dependent structural dysfunction of the NMJ. A *Drosophila* screen for altered synaptic structure in larvae found that the chromatin insulator *bpd* [also known as *mod(mdg4)*] was necessary for proper NMJ formation (Gorczyca et al., 1999). Non-lethal mutants of the gene were found to harbor additional axonal branch points, and boutons failed to form tight clusters. *Bpd* is a chromatin factor that regulates transcriptional silencing by binding DNA sequences known as *gypsy* insulators (Gerasimova et al., 1995, 2000; Chen and Corces, 2001). A screen for memory formation mutants also identified the gene as well as a later microarray screen for mushroom body-specific genes (Dubnau et al., 2003; Kobayashi et al., 2006). Taken together, these results imply this epigenetic regulator as necessary for proper synaptic structure formation. Notably, chromatin insulation is thought to become disrupted with age (Fu et al., 2008). Despite this, the exact genes regulated by *Bpd* in a neuronal context remain unknown.

### Epigenetic Factors can Directly Modify Synaptic Proteins

A series of recent studies on the larval NMJ revealed that deacetylases may also play non-canonical roles in alterations of presynaptic active zone morphology. The protein ELP3 is a histone acetyl-transferase (HAT) that was shown to be able to acetylate the synaptic *t*-bar protein Bruchpilot (Brp) and thereby promote its degradation (Han et al., 2008; Miśkiewicz et al., 2011). Intriguingly, another histone-interacting protein, histone deacetylase 6 (HDAC6) was also found to regulate Brp acetylation in opposite fashion to ELP3 (Miskiewicz et al., 2014). These results imply that proteins involved in epigenetic regulation may have additional functions at the presynaptic terminal. Epigenetic changes in the nucleus may promote the shuttling of these proteins between the nucleus and cytoplasm. A different epigenetic regulator, HDAC4, changes its cellular localization in response to neuronal activity (Chawla et al., 2003). It is currently not known how the intracellular localization of these chromatin-modifying proteins changes with age.

## Epigenetics of Synaptic Function and Aging

### Plasticity is Altered by Aging

While the structure of the synapse clearly contributes to its proper function, failure of the neuron to properly maintain and adjust neurotransmitter release is possibly even more deleterious and wide-spread. The formation of memories requires synaptic plasticity in the form of Hebbian (“fire together, wire together”) synaptic facilitation, long-term potentiation (LTP), and long-term depression (LTD). Defects in LTP induction and maintenance with age have been correlated to declines in memory and cognitive ability (Penner et al., 2010). The extent of the effect of aging on the *induction* of LTP seems to depend on anatomical region and stimulation paradigm (Rosenzweig and Barnes, 2003). *Maintenance* of LTP, on the other hand, has been robustly shown to decrease with age (Landfield et al., 1978; Barnes, 2003). Studies have found that the slowing of aging by caloric restriction also preserved LTP maintenance in aged rats (Hori et al., 1992; McGahon et al., 1999; Eckles-Smith et al., 2000). Induction of LDP by low frequency stimulation in CA1 neurons of aged rats was able to reliably and rapidly reverse LTP, in contrast to what is observed in the neurons of younger rats (Norris et al., 1996).

Other forms of synaptic plasticity may also become disrupted with age. Non-Hebbian homeostatic mechanisms are believed to be required in neural networks to prevent a cascade of positive-feedback induced hyperactivity (Turrigiano, 2011). Homeostatic synaptic scaling has not been studied in the context of normal aging, however there is evidence it is impaired with age from studies of neurodegenerative disease. For example, in a mouse model of AD (with a double knock-in of presenilin 1 and amyloid precursor protein) AMPA receptor particles decreased in old age (Chang et al., 2006). There was also a downscaling of AMPA receptor mediated currents after middle age. This in turn led to defects in LTD and LTP. Despite a lack of change in control mice, this suggests that there is an age-component to the defects of homeostatic scaling seen in AD model mice. It is possible that these changes may mimic homeostatic dysfunctions that happen at very advanced ages.

Although many laboratories have studied the effects of pathological and non-pathological aging on the functions of the *Drosophila* nervous system using behavioral paradigms, the effects of non-pathological aging on synapse function has not been thoroughly studied (Jones and Grotewiel, 2011). It is clear that *Drosophila* also undergo age-dependent declines in memory formation and maintenance (Tamura et al., 2003; Simon et al., 2006). This has been found to occur in a PKA-dependent manner (Yamazaki et al., 2007). Given that PKA signaling is an important for changing the post-synaptic membrane channel composition, it implies that aging is causing central synapse dysfunction in *Drosophila*. (Silva et al., 1998) In addition, studies investigating the propagation of signals through the giant fiber circuit have demonstrated age-dependent degradation of circuit function that could be due to changes in neurotransmission (Zhao et al., 2010).

A new study in the adult *Drosophila* NMJ found that there is an age-dependent change in the homeostatic set point of the innervating neuron (Mahoney et al., 2014). Although the release properties of the cell are stable across 7 and 35 days of age, in 42-day-old animals the excitatory post-synaptic potential and quantal content (measures of synaptic vesicle release) consistently increases by approximately 70%. This shift was found to be distinct from canonical homeostatic signaling itself, as it was also seen in *ephexin* mutants (in which homeostasis is inhibited). This implies a change in cell-intrinsic properties with age-possibly an increase in active voltage-gated calcium channels or synaptic vesicle fusion proteins. The observed set-point change also has some potentially negative physiological consequences, as demonstrated by the enhanced synaptic depression observed at aged synapses. For an aged fly, this may cause motor behavior deficits in demanding environmental circumstances. This study is particularly intriguing because of the defined window during which there seems to be a shift in the properties of the innervating cell, potentially indicating wide changes in gene expression and neuronal identity.

### Histone Acetylation and Plasticity

Epigenetics has emerged as a key regulator of long-term memory formation. A seminal study in the *Aplysia* snail by the Kandel laboratory found that by applying a long-term facilitation (a presynaptic form of synaptic strengthening) inducer and an LTD inducer at different terminals of a bidirectionally branched neuron the response to these inputs was integrated at the nucleus by the factors CREB1 and CREB2. CREB1 activates the HAT CPB, whereas CREB2 recruits HDAC5. These two chromatin remodeling complexes compete at the promoter of the C/EBP transcription factor which regulates synaptic structure and strength. In the case studied, LTD was found to epigenetically dominate facilitation (Guan et al., 2002).

Vertebrate studies have also found that histone modification is necessary for plasticity. Mice deficient in methyl CpG binding protein (MBD1) were found to harbor defects in neurogenesis and LTP (Zhao et al., 2003). A landmark study by Levenson and colleagues showed that memory formation transiently increases the acetylation of histone H3, and that LTP induction can be increased by administering HDAC inhibitors to mice (Levenson et al., 2004). Other studies have demonstrated the importance of HATs on LTP and memory (Alarcón et al., 2004; Korzus et al., 2004). Behavioral assays also implicate a synaptic role for histone acetylation. In mice, significant age-dependent declines in fear conditioning and memory were observed and histone H4K5 acetylation levels were altered more slowly in old mice. Peleg et al. (2010) and other studies have found evidence supporting epigenetic dysregulation in cognitive aging. Knocking out HDAC6 in a mouse model of AD ameliorated cognitive defects in 8-month old mice (Govindarajan et al., 2013).

### CpG Methylation and Plasticity

Histone acetylation is not the only epigenetic mechanism that can affect synapse function. CpG methylation has been heavily implied in LTP maintenance. In VP16-CREB mice, which consolidate persistent LTP after weak stimulus, microarray comparisons to wild types revealed that brain derived neurotrophic growth factor (BDNF) was a key component (Barco et al., 2005). A study in rats found a similar result and also demonstrated that LTP maintenance could be abrogated by administration of DNMT and HDAC inhibitors (Sui et al., 2012). BDNF expression has previously been shown to be regulated by DNA CpG methylation (Martinowich et al., 2003). Recent studies have found that BDNF methylation is be required for fear memory consolidation, and can be altered in early life in mice by events such as early weaning or isolation (Lubin et al., 2008; Roth et al., 2009). Additionally, a recent study found that age-dependent changes to BDNF expression could be reversed by epigenetic manipulation (Zeng et al., 2011).

In aged rats with differential cognitive decline outcomes (that is, a population of the same age but with individuals of different cognitive capacities), individuals with less decline in cognition were shown to use an alternative LTD mechanism that was NMDA receptor–independent (Lee et al., 2005). Within-population variability suggests that epigenetic differences among individuals could play a role in aging outcomes. One potential source of variation among aged individuals is CpG methylation, which has been shown to diverge with age, even in isogenic backgrounds (Fraga et al., 2005; Kaminsky et al., 2009).

Intrinsic homeostatic plasticity has also been shown to be epigenetically regulated. Recent work has shown that phosphorylation of methyl-CpG binding protein 2 (MeCP2) was required for synaptic scaling in mouse hippocampal neurons (Zhong et al., 2012). Mutations in MeCP2 had previously been identified as causing the autism-spectrum disorder Rhett’s syndrome (Amir et al., 1999). The authors mutated the serine residues in MeCP2 that were phosphorylation targets to alanine residues and found that synaptic downscaling induced by bicuculline was disrupted, but not up scaling by tetrodotoxin (TTX). Together, these findings support CpG methylation deregulation as contributing to age-related decline in synaptic plasticity and homeostasis.

## Concluding Remarks

If defects at the synapse are proximally caused by epigenetic dysregulation of key synaptic genes, then what are the distal causes that drive neuronal epigenetic dysfunction with age? One possibility is that changes in epigenetic regulation are the unavoidable consequence of species-specific noise in global transcription. Gene expression is not perfectly regulated and has large intrinsic stochasticity (Kaern et al., 2005). This would help explain why genetically identical conspecifics of the same chronological age can have dramatically different mortality risk and health. Aging-accumulated changes in expression of epigenetic regulators may cascade into larger changes culminating with alterations in cell function and identity. Synapses would be particularly sensitive to these disruptions.

**Figure 1:**
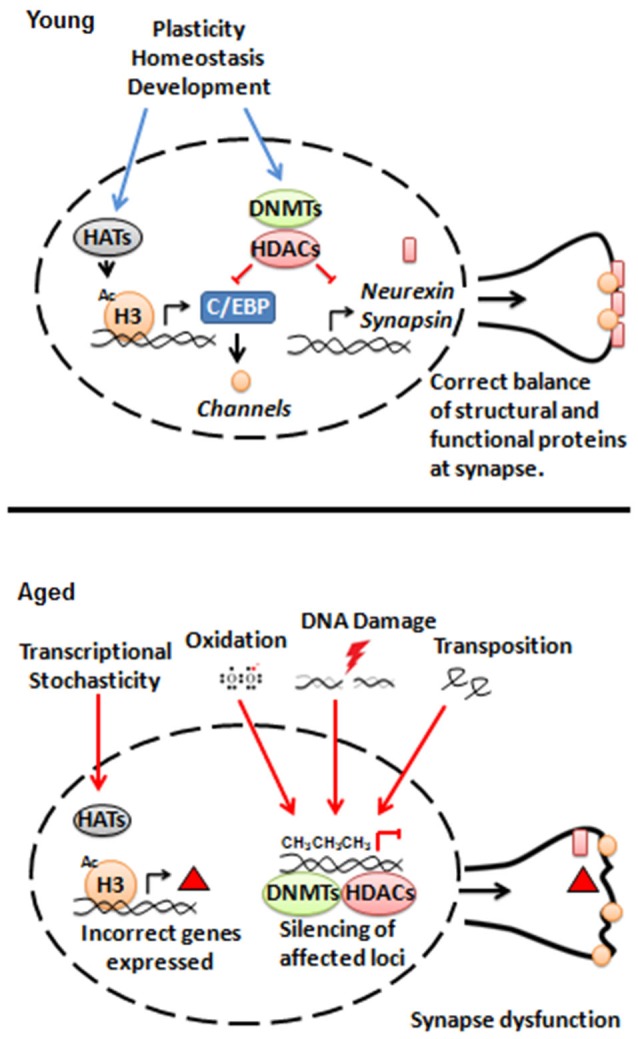
**Model for interactions between epigenetic regulators and synaptic structure and function during aging**.

Another possibility is that an insult or damage that normally accumulates with age (e.g., oxidation, advanced age glycation products, DNA damage, amyloid aggregation, etc.) gradually interferes with normal epigenetic control. DNA lesions are a particularly interesting candidate, as their repair requires histone remodeling and the epigenetic marks may not be fully reverted to their original state following repair (Polo et al., 2006; Groth et al., 2007). DNA damage can also create opportunities for retrotransposon activation (Van Meter et al., 2014) and insertion, which can potentially disrupt the epigenetic state of surrounding chromatin (Farkash and Luning Prak, 2006). Evidence for transposable element activity increasing with age has been reported in *Drosophila* neurons (Li et al., 2013) and more generally in mammalian neurons (Erwin et al., 2014). A sketch of these possibilities is shown in Figure 1.

Most regulatory hierarchies remain unknown, and the epigenetic markup of many tissues is still understudied. The nervous system has received special attention in this regard given the prerequisite for memory formation on dynamic adjustment of gene expression. Epigenetic marks and proteins have also received attention from those working in the aging field, ever since the discovery that HDACs and sirtuins could modulate lifespan. We suggest that a more thorough synthesis of the two fields is warranted.

## Conflict of Interest Statement

The authors declare that the research was conducted in the absence of any commercial or financial relationships that could be construed as a potential conflict of interest.
